# The Gut Microbiome Obesity Index: A New Analytical Tool in the Metagenomics Workflow for the Evaluation of Gut Dysbiosis in Obese Humans

**DOI:** 10.3390/nu17142320

**Published:** 2025-07-14

**Authors:** Maria Kulecka, Paweł Jaworski, Natalia Zeber-Lubecka, Aneta Bałabas, Magdalena Piątkowska, Paweł Czarnowski, Barbara Frączek, Wiesław Tarnowski, Michał Mikula, Jerzy Ostrowski

**Affiliations:** 1 Department of Gastroenterology, Hepatology and Clinical Oncology, Centre of Postgraduate Medical Education, 02-781 Warsaw, Poland; mkulecka@cmkp.edu.pl (M.K.); natalia.zeber-lubecka@cmkp.edu.pl (N.Z.-L.); 2 Department of Genetics, Maria Sklodowska-Curie National Research Institute of Oncology, 02-781 Warsaw, Polandmichal.mikula@nio.gov.pl (M.M.); 3 Department of General, Oncological and Bariatric Surgery, Centre of Postgraduate Medical Education, Orłowski Hospital, Czerniakowska 231, 00-416 Warsaw, Poland; 4 Department of Sports Medicine and Human Nutrition, Institute of Biomedical Sciences, University of Physical Education, 31-571 Krakow, Poland

**Keywords:** obesity, microbiome, metabolome, health index, dysbiosis

## Abstract

**Background/Objectives**: Our aim was to create a new method for analyzing metagenomics data, named the gut microbiome obesity index, using a set of taxa/biological functions that correlated with BMI. **Methods**: A total of 109 obese patients (73 women and 36 men, median BMI 43.0 kg/m^2^), 87 healthy control (HC) individuals (39 females and 48 males, median BMI 22.7 kg/m^2^), and 109 esports players (five females and 104 males, median BMI 23.0 kg/m^2^) were included in the study. To conduct metagenomic and metabolomic analyses, DNA and selected metabolites were isolated from fecal samples and used for whole-genome shotgun sequencing and gas chromatography/mass spectrometry, respectively. **Results**: Compared with HCs and esports players, obese patients with a BMI > 40 kg/m^2^ had a significantly higher alpha diversity, as analyzed by the Shannon index, and significant dissimilarities in beta diversity. Both richness and diversity measures were correlated with BMI. Compared with HCs and esports players, 12 differential bacteria were found in the overall obesity group and 42 were found in those with a BMI > 40 kg/m^2^. Most of the altered species belonged to the *Lachnospiraceae* family. When the logarithmic relationship of the sums of the bacteria correlated with BMI was calculated to establish a taxonomic health index, it better differentiated between the obesity groups than a standard analytical pipeline; however, it did not differentiate between the HC and the BMI < 35 kg/m^2^ obesity group. Therefore, we created a functional index based on BMI-associated biological pathways, which differentiated between all obesity groups. **Conclusions**: Of the obesity indices used to distinguish between healthy and obese microbiota analyzed in this study, a function-based index was more useful than a taxonomy-based index. We believe that gut microbiome indexes could be useful as part of routine metagenomics evaluations. However, an index developed in one geographical area might not be applicable to individuals in a different region and, therefore, further studies should develop separate indices for different populations or geographical regions rather than relying on a single index.

## 1. Introduction

Obesity in adults is defined by a BMI of 30 kg/m^2^ or greater [1] and presents a significant global health concern. In 2020, the number of obese individuals reached nearly one billion, approximately 14% of the global population. This resulted in the economic burden of elevated BMI (≥25 kg/m^2^) reaching an alarming figure of almost two trillion US dollars [2]. Although historically concentrated in industrialized, developed countries, recent research indicates a growing prevalence of obesity in low-income and developing countries [3,4,5]. Class II (BMI ≥ 35 and <40 kg/m^2^) and III obesity (BMI ≥ 40 kg/m^2^) are particular public health concerns because of their consistent association with increased risks of comorbidities such as type 2 diabetes, hypertension, and cardiovascular disease [3,6].

In recent years, the association between gut microbiota and obesity has gained increasing attention. The composition and function of gut microbiota are modulated by factors including host genotype, age, sex, lifestyle, diet, physical activity, and sanitation [7]. Intestinal microbial dysbiosis can affect appetite, energy absorption, fat storage, and circadian rhythm through a complex network of host–microbe interactions [8]. However, the role of gut microbiome changes in the development of obesity has not yet been defined. An increased *Firmicutes*/*Bacteroidetes* ratio is often cited as a potential biomarker for obesity (reviewed in [9]), although recent meta-analyses (Walters et al. [10] and Sze et al. [11]) did not validate this association. Moreover, these studies revealed inconsistent associations between the diversity of gut microbiota and obesity. Alpha diversity, evaluated by the Shannon index, is a marker of bacterial richness and evenness. This marker was significantly lower, higher, and not significantly different in obese adults compared with non-obese adults in nine, two, and 11 studies, respectively. In addition, the Chao1 estimator for richness was lower in obese individuals than non-obese individuals in three studies, higher in two studies, and not significant in two studies, according to a recent systematic review and meta-analysis [12].

At the genus level, a lower abundance of Bifidobacterium and Eggerthella and a higher abundance of Acidaminococcus, Anaerococcus, Catenibacterium, Dialister, Dorea, Escherichia-Shigella, Eubacterium, Fusobacterium, Megasphaera, Prevotella, Roseburia, Streptococcus, and Sutterella were found in obese people than in non-obese people, while Akkermansia, Alistipes, Anaerotruncus, Bacteroides, Blautia, Clostridium, Coprococcus, Desulfovibrio, Faecalibacterium, Oscillibacter, Oscillospira, Parabacteroides, and Ruminococccus were found in higher abundance in obese people in some studies and in lower abundance in others [12]. Functional alterations linked to obesity primarily affect short-chain fatty acid (SCFA) and bile acid metabolism [13]. Another significant disturbance associated with the gut microbiome in obesity is metabolic endotoxemia. This is characterized by a high-fat diet-induced elevation of plasma lipopolysaccharide, which leads to chronic inflammation in obese individuals [14].

As considered previously [15], the results of metagenomics studies are highly dependent on the analytical pipeline used, which can introduce bias to the results. For example, there may be variations in the proportion of reads used or the selection of richness and diversity indices and individual taxa for analysis; therefore, inconsistencies between the results of previous studies are not surprising. As a result, when analyzing high-throughput sequencing data, bioinformatic pipeline selection is a critical step in study design [16]. Moreover, single taxa or diversity measures alone are insufficient as markers of obesity. As an alternative, dysbiosis indices have been proposed to characterize microbiota disturbances associated with specific conditions (reviewed in [17]); these are based primarily on combinations of specific taxa [18,19]. Of these indices, the Gut Microbiome Health Index (GMHI) [19], which is based on the ratio of 50 microbial species associated with healthy or unhealthy gut ecosystems, exceeds 73% accuracy in determining a disease state. The Gut Microbiome Wellness Index [20] is based purely on gut taxonomic signatures. The hiPCA [21] uses the contribution of highly influential (ostensibly keystone) species in different groups of patients. Other analytical methods employ the lasso penalized logistic regression model [20] and random forest-based machine learning classifiers [22]. While all of these are dependent on taxonomic classification, Zielińska et al. [23] have proposed an index based on functional characteristics of the gut microbiome.

With access to stool samples from people with a wide range of BMIs, we analyzed metagenomics data using a standard analytical pipeline previously employed in our research. The results obtained allowed us to create a new method of analysis, named the gut microbiome obesity index, using a set of taxa and pathways whose relative abundances correlated with BMI.

## 2. Materials and Methods

### 2.1. The Participants

A total of 305 participants were enrolled in this study, including 109 obese patients, 87 healthy control individuals, and 109 esports players. None of the participants had used antibiotics in the 2 months before fecal sampling or had inflammatory bowel disease or a history of cancer. The obese patients (73 women and 36 men; median age, 43 years, range, 18–62 years; median BMI, 43.0 kg/m^2^, range 32.0–77.1 kg/m^2^) were recruited mostly from those who qualified for bariatric surgery at the Department of General, Oncological, and Bariatric Surgery (Centre of Postgraduate Medical Education, Orłowski Hospital, 00-416 Warsaw, Poland). The control group consisted of 39 females and 48 males (median age, 32 years, range, 18–62 years; median BMI, 22.7 kg/m^2^, range, 18.0–28.7 kg/m^2^) and the esports player group consisted of five females and 104 males (median age, 21 years, range 16–21 years; median BMI, 23.0 kg/m^2^, range, 15.1–32.1 kg/m^2^). The participants in the esports player group who declared being in good health remained on a Western-type diet without specific dietary restrictions and led a sedentary lifestyle.

This study was conducted in accordance with the principles of the Declaration of Helsinki and was approved by the Maria Sklodowska-Curie National Research Institute of Oncology Local Bioethics Board (decision 54/2017) (40/2018/1/2019). All participants provided informed consent to participate.

### 2.2. Metagenomics Analysis and Metabolite Profiling

Metagenomics and metabolomics were performed as described previously [15] using self-collected frozen fecal samples. Metagenomic sequencing was conducted on the Illumina NovaSeq 6000 platform (San Diego, CA, USA), and selected SCFAs and amino acids were detected on an Agilent 7000D Triple Quadrupole mass spectrometer coupled to a 7890 GC System with a G4513A autosampler (Agilent Technologies, Santa Clara, CA, USA).

### 2.3. Bioinformatics and Statistical Analyses

#### 2.3.1. Bacteria and Metabolites

KneadData version 0.10.0 (with default parameters) was used to quality trim sequences and remove sequences originating from the host. Bacterial taxa were identified using Kraken2 [24] version 2.1.3 with default databases and parameters, with the exception of confidence, which was set to 0.1. Species-level assignments were then performed with Bracken version 2.7 using a minimum of 100 counts. The processing pipeline was made public under 10.5281/zenodo.15801557. Shannon diversity indices were computed with the iNEXT package [25] version 3.0.0, using default parameters.

For differences in taxa abundance between groups the LinDA (Linear (Lin) Model for Differential Abundance (DA)) [26] method for compositional data was applied, with *p*-values corrected using the Benjamini–Hochberg [27] procedure to minimize the false discovery rate. For diversity indices, their values were compared globally using the Kruskal–Wallis test and between two groups using the Mann–Whitney U-test. In metabolite analysis, differences in concentrations ad relative between two groups were assessed using the Mann–Whitney U-test. Both tests were two-sided.

#### 2.3.2. Bacteria and Metabolite Associations

Non-ambiguous associations between taxa and at least one metabolite were identified with the metadeconfoundR [28] package version 0.3. For this analysis, only taxa which had more than a thousand assigned reads on average and were present in at least 10% of samples were kept. Following the mixOmics [29] version 6.22 tutorial (http://mixomics.org/case-studies/rcca-nutrimouse-case-study/, accessed on 23 November 2024), regularized canonical correlation analysis was applied on identified taxa and metabolites using the ridge method with parameter tuning. The correlation structure was plotted with the complexHeatmap [30] package version 2.14. Bacterial species were divided into clusters using Ward’s method (the “ward.D2” method in the base R hclust function). The optimal number of modules was chosen using the dynamicTreeCut [31] package version 1.63.

Functional assignments were performed using human version 3.0 (part of BioBakery Workflows) [32], with MetaCyc [33] pathways as a reference database. KneadData version 0.10 was applied for quality filtering and decontamination prior to this analysis. The LinDA method was used to identify differential abundance in pathway data, with *p*-values corrected using the Benjamini–Hochberg procedure to minimize the false discovery rate.

#### 2.3.3. Index Computation

Taxonomic and functional indices were calculated as previously described [15] using bacteria/pathways that were significantly positively or negatively associated with BMI (after correcting for age and sex) as nominators and denominators, respectively. External indices were calculated according to the method described by Zielińska et al. [23], using functional annotations only, as well as a mixture of functional and taxonomic annotations. Receiver operating curves (ROCs) and areas under them (AUCs) were calculated using the pROC R package [34].

## 3. Results

This study investigated 305 participants, of which 109 were obese patients, 87 were healthy control individuals, and 109 were esports players. The healthy control group provided a baseline of what is considered normal or typical in terms of markers of health, physiological responses, and physical performance. The group of esports players constituted another reference group that differed significantly from healthy individuals. Esports players, despite normal BMI, have a sedentary lifestyle and follow a Western diet, which can potentially introduce some metabolic confounders.

DNA isolated from fecal samples was analyzed using whole-genome shotgun metagenomic sequencing. On average, 15 million reads were generated per sample (median, 13 million). Six out of the 24 identified phyla had an abundance equivalent to more than 1% of the microbiome (*Bacteroidota*, *Bacillota*, *Actinomycetota*, *Pseudomonadota*, *Verrucomicrobiota*, and *Uroviricota*). Five bacterial phyla accounted for more than 98% of reads. Our dataset contained 193 species that were present in more than 0.01% of reads (out of 1130 species detected). Out of these, the ten most abundant species belonged to the *Phocaeicola/Bacteroides* and *Alistipes* genera.

### 3.1. Bacterial Diversity and Composition

**Figure 1:**
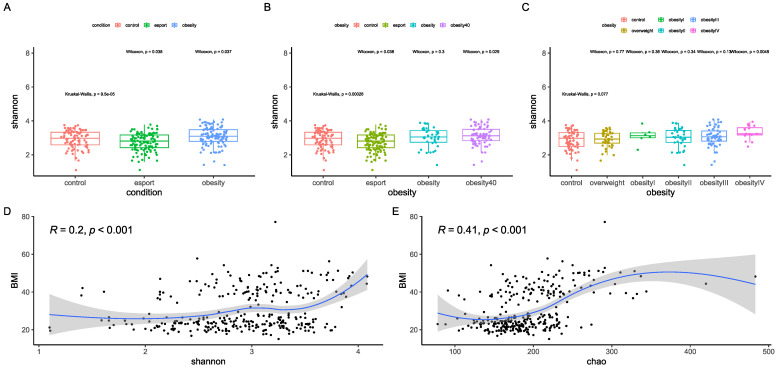
Differences in an alpha-diversity index (Shannon index, (**A**–**C**)) between obesity and control groups. For image (**C**), the strata were as follows: an overweight group (BMI > 25 and ≤30 kg/m^2^, n = 41), obesity I (BMI > 30 and ≤35 kg/m^2^, n = 6), obesity II (BMI > 35 and ≤40 kg/m^2^, n = 33), obesity III (BMI > 40 and ≤50 kg/m^2^, n = 53), and obesity IV (BMI > 50 kg/m^2^, n = 17). The correlation between BMI and diversity (Shannon index; (**D**)) and richness (Chao1 index; (**E**)), as calculated by Spearman’s rank correlation coefficient. Sequencing depth was at 15 million reads/sample (Illumina NovaSeq 6000) for metagenomics. Statistical tests used were Kruskal–Wallis and Mann-Whitney U (pairwise comparison).

Alpha diversity was analyzed using the Shannon index, a marker of bacterial richness and evenness, and beta diversity was analyzed using principal component analysis of Aitchison distances. While alpha diversity was slightly but significantly lower in the esports group than in the control group, it was significantly higher in the obesity group (both *p* < 0.05; Figure 1A). To investigate this further, we split the obesity group in two, using a BMI of 40 kg/m^2^ as the threshold. This time, only the group with a BMI higher than 40 kg/m^2^ had a significantly higher alpha diversity than the control group (*p* < 0.05; Figure 1B). Next, we stratified participants into a control group (BMI ≤ 25 kg/m^2^, n = 154), an overweight group (BMI > 25 and ≤30 kg/m^2^, n = 41), and four obesity groups: obesity I (BMI > 30 and ≤35 kg/m^2^, n = 6), obesity II (BMI > 35 and ≤40 kg/m^2^, n = 33), obesity III (BMI > 40 and ≤50 kg/m^2^, n = 53), and obesity IV (BMI > 50 kg/m^2^, n = 17). Only the highest obesity group had significantly higher diversity than normal-weight controls (*p* < 0.01; Figure 1C). We also investigated the correlation between BMI and the Shannon index, as well as Chao1, an index that accounts for bacterial richness only. Both indices were significantly correlated with BMI (both *p* < 0.001; Figure 1D,E), although Chao1 had a higher correlation coefficient.

### 3.2. Beta Diversity

**Figure 2:**
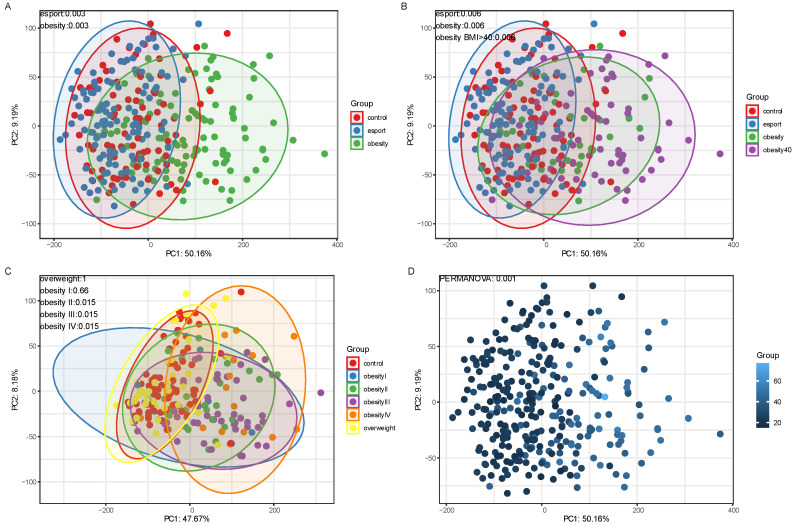
(**A**–**C**) Principal component (PC) analyses of differences in beta diversity between control and obesity groups, as measured by PERMANOVA tests on Aitchison distances. For image (**C**), the strata were as follows: an overweight group (BMI > 25 and ≤30 kg/m^2^, n = 41), obesity I (BMI > 30 and ≤35 kg/m^2^, n = 6), obesity II (BMI > 35 and ≤40 kg/m^2^, n = 33), obesity III (BMI > 40 and ≤50 kg/m^2^, n = 53), and obesity IV (BMI > 50 kg/m^2^, n = 17). (**D**) Association with beta diversity and BMI. Sequencing depth was at 15 million reads/sample (Illumina NovaSeq 6000) for metagenomics.

Individuals in both the esports and obesity groups had significantly different beta diversities than the control group (both *p* < 0.001; Figure 2A). When the obese group was separated into individuals with a BMI < 40 kg/m^2^ and those with a BMI > 40 kg/m^2^, both still had beta diversities significantly different from the control group (*p* < 0.01; Figure 2B). Further stratification revealed that the obesity groups II–IV displayed significant dissimilarities compared with the control group (all *p* < 0.001; Figure 2C). BMI gradient was also significantly associated with dissimilarity (Figure 2D) and was significantly correlated with the first principal component (Spearman’s rank correlation coefficient r = 0.52, *p* < 0.001).

### 3.3. Differential Abundance

**Figure 3:**
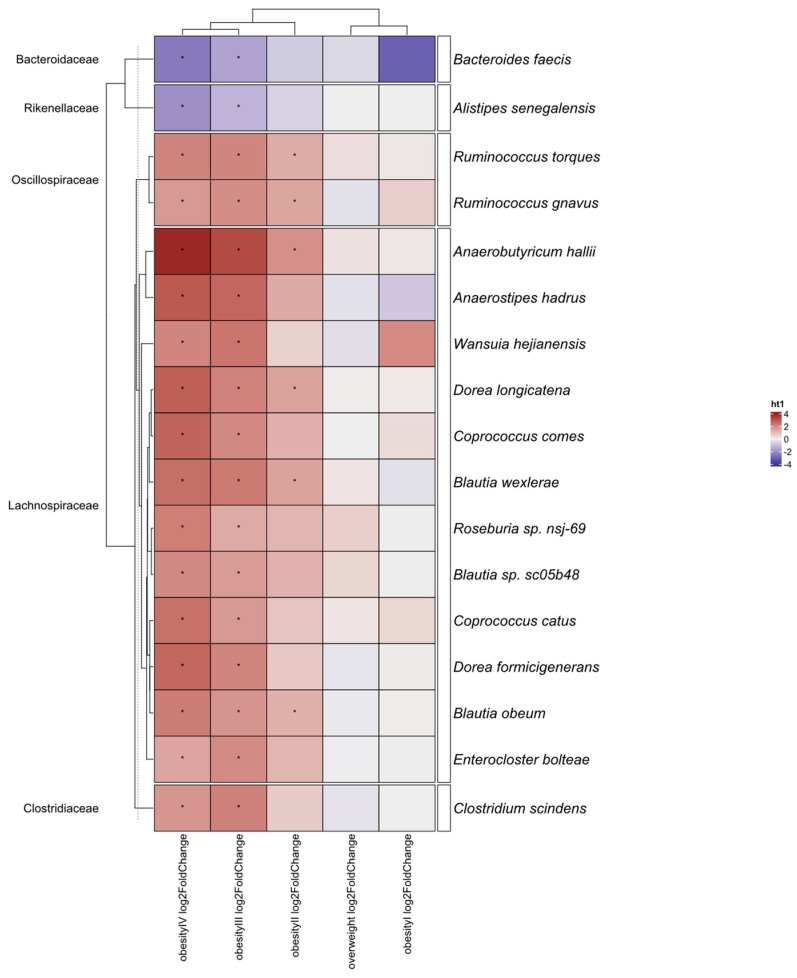
Bacteria commonly altered in obesity groups compared with the control group. Stars indicate significant differences in taxon abundance between an obesity group and the control group (*p* < 0.05). Statistical method used: LinDA with FDR correction. The strata were as follows: an overweight group (BMI > 25 and ≤30 kg/m^2^, n = 41), obesity I (BMI > 30 and ≤35 kg/m^2^, n = 6), obesity II (BMI > 35 and ≤40 kg/m^2^, n = 33), obesity III (BMI > 40 and ≤50 kg/m^2^, n = 53), and obesity IV (BMI > 50 kg/m^2^, n = 17). Sequencing depth was at 15 million reads/sample (Illumina NovaSeq 6000) for metagenomics.

In testing for differential abundance, we followed the same stratification process as for alpha and beta diversity. For comparison between the control and esports groups, only two differential species were identified: *Coprococcus eutactus* and *Simiaoa sunii*. By contrast, 39 species differentiated healthy controls from the obesity group; of these, 28 were overrepresented in obese individuals (Supplementary Table S1). These included the mucin degraders [35] *Akkermansia muciniphila* and *Ruminococcus torques*, as well as bacteria associated with biochemical parameters such as elevated triglycerides [36] (*Eggerthella lenta* and *R. torques*) and high-density lipoprotein cholesterol (*Coprococcus catus*). Further stratification revealed 12 differential bacteria for obesity and 42 for extreme obesity (BMI > 40 kg/m^2^). Differential bacteria common in both comparisons included *Escherichia coli, E. lenta, R. torques*, and *Blautia obeum*, while *Akkermansia muciniphila* and pathogens (*Clostridioides difficile* and *Enterocloster bolteae*) were observed in the BMI > 40 kg/m^2^ group only. In the final stratification, no significant differences were observed between the control group and the overweight and obesity I groups. Small differences were visible for the obesity II group (six species), while the obesity III and IV groups exhibited larger differences (25 and 23 species, respectively). Every species altered in the obesity II group was also altered in the higher BMI classes. Most of the altered species belonged to the *Lachnospiraceae* family (Figure 3, Supplementary Table S2).

### 3.4. Metabolite Correlations

**Figure 4:**
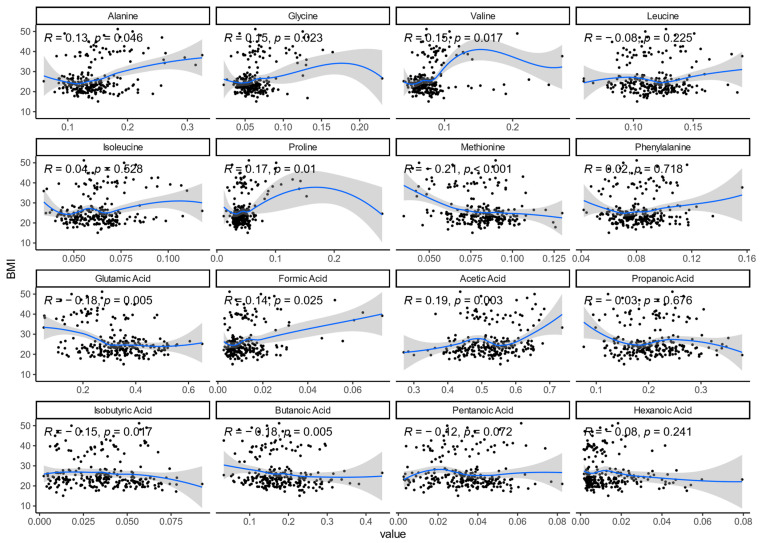
Correlations between BMI and amino acids and short-chain fatty acids percentages, as measured by Spearman’s rank correlation coefficient.

We also tested associations between obesity and a select group of metabolites, mainly SCFAs and amino acids. In terms of relative abundance of metabolites, the obese group had a significantly higher percentage of formic acid than both the control and esports groups. Statistically significant differences were observed for all amino acids except leucine and phenylalanine (Supplementary Table S3). The results were similar if the obesity group was further separated into individuals with a BMI < 40 kg/m^2^ and those with a BMI > 40 kg/m^2^, except for isoleucine (significant only for the BMI > 40 kg/m^2^ group) and methionine (significant only for the BMI < 40 kg/m^2^ group, Supplementary Table S4). Interestingly, further stratification revealed differences between the overweight and control groups in formic acid relative abundance (Supplementary Table S5). Most metabolites were significantly correlated with BMI (Figure 4, Spearman’s rank correlation coefficient *p*-value < 0.05); in addition, except for methionine, all correlation coefficients were between −0.2 and 0.2.

### 3.5. Associations Between Bacteria, BMI, and Metabolites

**Figure 5:**
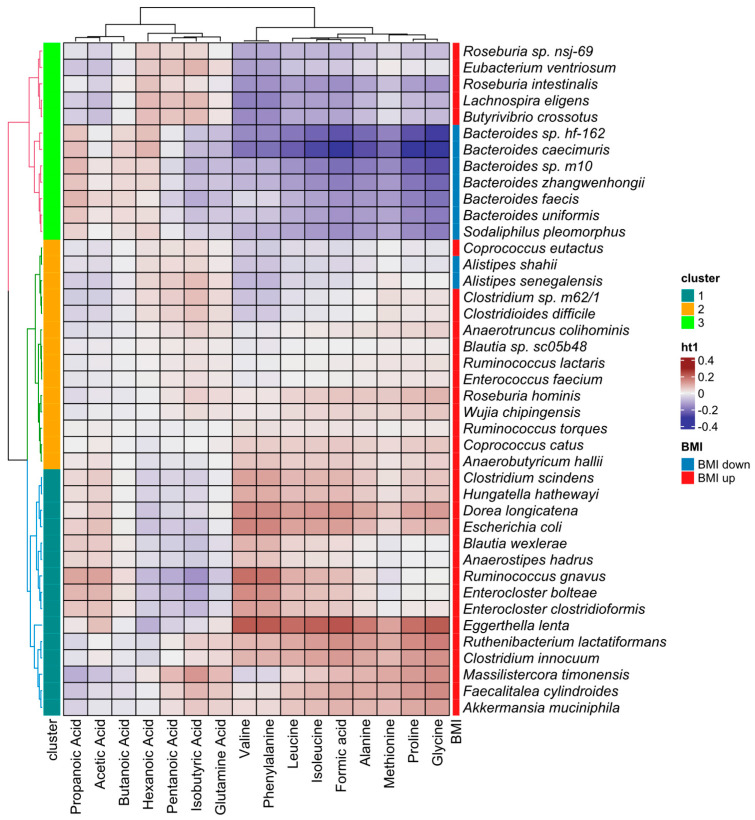
Correlations between metabolites and bacteria that correlated significantly with BMI, according to the first and second canonical variates from regularized canonical correlation analysis.

We also used the LinDA method to test whether any bacteria had a linear relationship with BMI. We found 48 such species (padj < 0.05), most of which (except for nine bacteria, mainly from the *Alistipes* and *Bacteroides* genera, Supplementary Table S6) had higher abundance at a higher BMI. Among taxa positively correlated with BMI were bacteria previously associated with elevated high-density lipoprotein cholesterol levels, such as *Coprococcus catus* and *Coprococcus eutactus*, as well as butyrate producers such as *Roseburia intestinalis*, *Roseburia hominis*, and *Anaerostipes hadrus*. Regularized canonical correlation analysis revealed that 41 of these bacteria had significant correlations with metabolites (taking into account the first two canonical variates) and formed three distinct correlation clusters. The first cluster contained bacteria strongly positively correlated with amino acids and BMI. The second cluster contained bacteria mostly correlated positively with BMI, with the exception of the *Alistipes* genus. The third cluster contained bacteria from the *Bacteroides* genus, which were negatively correlated with BMI and amino acids and positively correlated with most SCFAs. However, members of this cluster also included bacteria positively correlated with BMI, mainly from the *Roseburia* genus (Figure 5).

### 3.6. Functional Analysis

**Figure 6:**
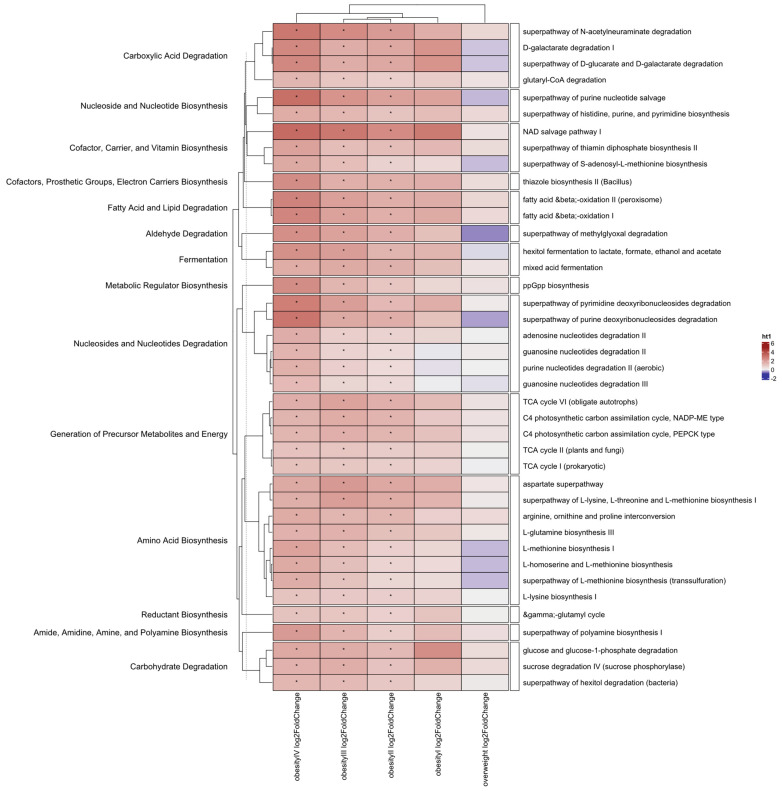
Pathways commonly altered when comparing control and obesity groups. Stars indicate significant differences in biological pathway abundance between an obesity group and the control group (*p* < 0.05). Statistical method used: LinDA with FDR correction. The strata were as follows: an overweight group (BMI > 25 and ≤30 kg/m^2^, n = 41), obesity I (BMI > 30 and ≤35 kg/m^2^, n = 6), obesity II (BMI > 35 and ≤40 kg/m^2^, n = 33), obesity III (BMI > 40 and ≤50 kg/m^2^, n = 53), and obesity IV (BMI > 50 kg/m^2^, n = 17). Sequencing depth was at 15 million reads/sample (Illumina NovaSeq 6000) for metagenomics.

Shotgun sequencing allows not only taxonomic insight but also functional insight into the microbiome. We therefore decided to compare the abundance of specific biological pathways in our participants. In the comparison between controls, esports players, and obese individuals, minor differences were observed between the esports and control groups (five pathways), which mostly concerned methionine biosynthesis. Interestingly, all pathways were present in lower abundance in the esports group. For the obesity group, major changes affecting 102 pathways were observed. After splitting the obesity group into two, 103 pathways were significantly different between the BMI > 40 kg/m^2^ group and the control group, while 40 pathways were significantly different between the BMI < 40 kg/m^2^ group and the control group; all differential pathways identified in the BMI < 40 kg/m^2^ group were common to both groups. Upon further stratification, no functional differences were found for the overweight and obesity I groups, while 41, 96, and 79 differential pathways were found for the obesity II, III, and IV groups, respectively. Of these, 40 pathways were shared across groups, including pathways associated with fatty acid oxidation and fermentation, as well as glucose and sucrose degradation (Figure 6).

### 3.7. Health Indices

**Figure 7:**
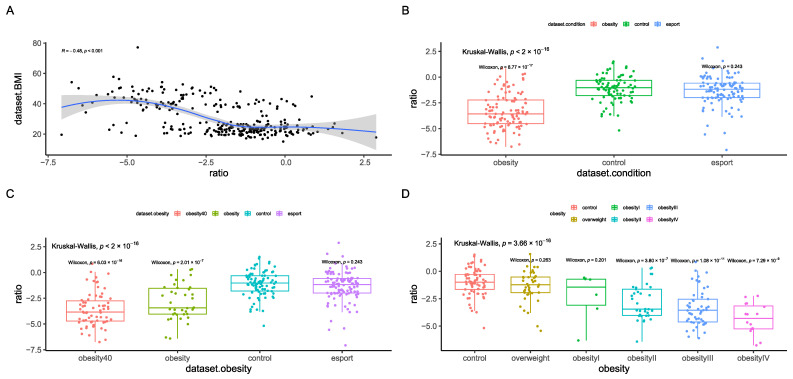
Taxonomic health index results, including correlation with BMI (**A**) according to Spearman’s rank correlation coefficient and differences between the control group and esports, overweight, and obesity groups (**B**–**D**). Statistical tests used were Kruskal–Wallis and Mann-Whitney U (pairwise comparison). The strata were as follows: an overweight group (BMI > 25 and ≤30 kg/m^2^, n = 41), obesity I (BMI > 30 and ≤35 kg/m^2^, n = 6), obesity II (BMI > 35 and ≤40 kg/m^2^, n = 33), obesity III (BMI > 40 and ≤50 kg/m^2^, n = 53), and obesity IV (BMI > 50 kg/m^2^, n = 17). Sequencing depth was at 15 million reads/sample (Illumina NovaSeq 6000) for metagenomics.

Finally, we sought to design a single index to differentiate between BMI groups. The first index we designed was a taxonomic index, which included bacteria that were significantly associated with BMI after correction for age and sex (Supplementary Table S7). As previously reported [15] we calculated the log ratio of the sums of bacteria positively and negatively correlated with BMI. This index correlated well with BMI (Figure 7A) and differentiated between the obesity groups (Figure 7B,C); however, it did not differentiate between the control and obesity I groups (Figure 7D).

**Figure 8:**
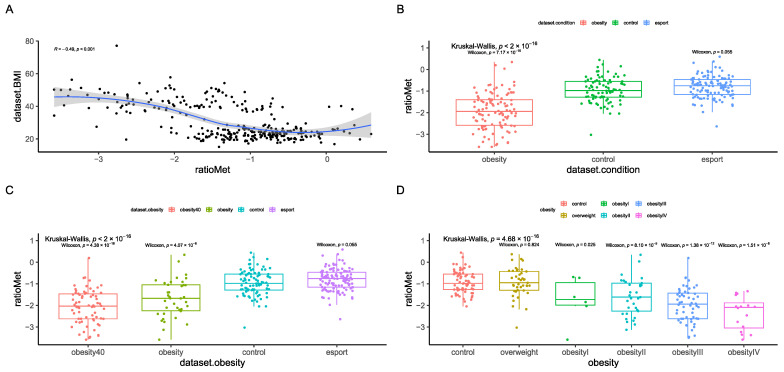
Functional health index results, including correlation with BMI (**A**) according to Spearman’s rank correlation coefficient and differences between the control group and esports, overweight, and obesity groups (**B**–**D**). Statistical tests used were Kruskal–Wallis and Mann-Whitney U (pairwise comparison). The strata were as follows: an overweight group (BMI > 25 and ≤30 kg/m^2^, n = 41), obesity I (BMI > 30 and ≤35 kg/m^2^, n = 6), obesity II (BMI > 35 and ≤40 kg/m^2^, n = 33), obesity III (BMI > 40 and ≤50 kg/m^2^, n = 53), and obesity IV (BMI > 50 kg/m^2^, n = 17). Sequencing depth was at 15 million reads/sample (Illumina NovaSeq 6000) for metagenomics.

Therefore, we created a functional index, which included pathways that were significantly associated with BMI after correction for age and sex (Supplementary Table S8). This index was significantly correlated with BMI (Figure 8A) and differentiated between the control and obesity groups (Figure 8B,C), including the obesity I group (Figure 8D).

**Figure 9:**
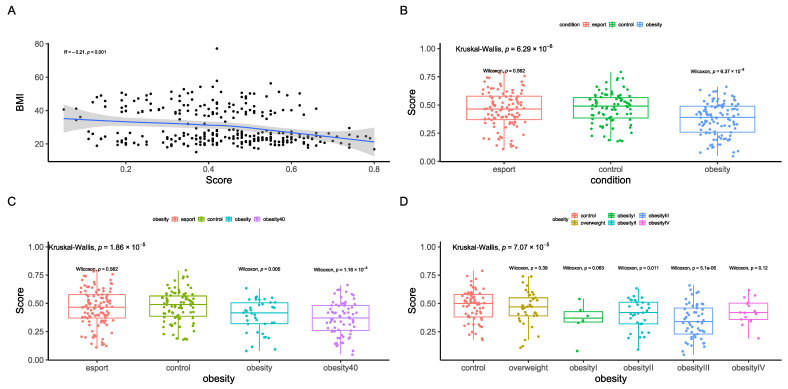
External functional health index (pHD) results, including correlation with BMI (**A**) according to Spearman’s rank correlation coefficient and differences between the control group and esports, overweight, and obesity groups (**B**–**D**). Statistical tests used were Kruskal–Wallis and Mann-Whitney U (pairwise comparison). The strata were as follows: an overweight group (BMI > 25 and ≤30 kg/m^2^, n = 41), obesity I (BMI > 30 and ≤35 kg/m^2^, n = 6), obesity II (BMI > 35 and ≤40 kg/m^2^, n = 33), obesity III (BMI > 40 and ≤50 kg/m^2^, n = 53), and obesity IV (BMI > 50 kg/m^2^, n = 17). Sequencing depth was at 15 million reads/sample (Illumina NovaSeq 6000) for metagenomics.

Finally, we tested the health score reported by Zielińska et al. [23] (pHD), which is based on functional annotation of the microbiome. This index had a weak but significant association with BMI (Figure 9A) and distinguished between the control and <40 kg/m^2^ or >40 kg/m^2^ obesity subgroups (Figure 9B,C). However, the differentiation was weaker for the obesity I and IV groups (Figure 9D, *p* > 0.05 according to the Kruskall–Wallis test). Another health score, which includes both functional and taxonomic parameters, did not differentiate between obesity groups and healthy controls (Supplementary Figure S1).

**Figure 10:**
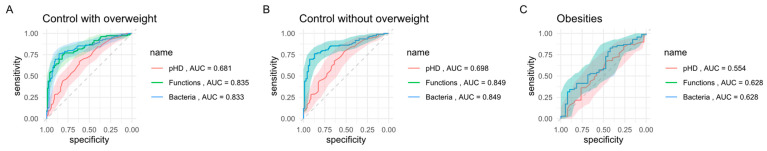
Receiver operating characteristic curves for classifying individuals into control and obesity groups (including overweight controls) (**A**), control and obesity groups (excluding overweight controls) (**B**), and different obesity groups (**C**). The compared indices are the index based on bacteria and functions as well as the pHD index. Areas under the curve (AUCs) for each receiver operating characteristic curve are given next to the legend descriptions.

According to receiver operating characteristic analyses, all the tested indices had good discriminative power for distinguishing between the control and obesity groups (all areas under the curve 0.681–0.849, Figure 10A,B). However, their performance was not as good at distinguishing between the BMI < 40 kg/m^2^ and BMI > 40 kg/m^2^ groups (areas under the curve 0.554–0.628, Figure 10C).

According to receiver operating characteristic analyses, all the tested indices had good discriminative power for distinguishing between the control and obesity groups (all areas under the curve 0.681–0.849, Figure 10A,B). However, their performance was not as good at distinguishing between the BMI < 40 kg/m^2^ and BMI > 40 kg/m^2^ groups (areas under the curve 0.554–0.628, Figure 10C).

## 4. Discussion

Experiments on germ-free mice [37,38,39] and accidental findings made after fecal microbial transfers in humans [40] first suggested that the microbiome might have a causative role in obesity. This made the gut microbiome both an attractive therapeutic target (reviewed in [41]) and a robust potential biomarker of obesity [42,43]. In line with earlier studies, we also found large differences in microbial signatures between normal-weight individuals and obese patients. However, these differences were mainly driven by people with higher levels of obesity (a BMI > 40 kg/m^2^). Specifically, these individuals were characterized by a significantly higher microbial diversity than normal-weight individuals. In addition, both species diversity and richness indices were positively correlated with BMI. While high diversity is often associated with gut microbial health [44], previous findings concerning obesity are ambiguous: while some studies suggest lowered microbial diversity in obesity [45,46], others report results to the contrary [47,48]; increased bacterial richness in obesity has also been reported [47,49]. Network analyses suggest that a high microbial diversity might indicate weaker microbiome stability [50] and negative microbial processes such as the bloom of transient colonizers [51]; in line with this, obese individuals are at higher risk of small intestine bacterial overgrowth [52]. Finally, negative associations between diversity and obesity might be specific to geographical regions: a meta-analysis suggested that this association was strongest among white Americans with high socio-economic status [53]. This suggests an overall bias in studies of the microbiome towards affluent, Western societies. Similar to our study, a previous study conducted in Poland is among those that reported increased diversity and richness in obese individuals [48].

The largest differences we observed on a species level were in people with a BMI > 40 kg/m^2^; very small differences were observed between the control and both the esports group and obese people with a BMI < 40 kg/m^2^. The most common differences were in bacteria from the *Lachnospiraceae* family, specifically *Blautia wexlareae* and *obeum*, *R. torques, Ruminococcus gnavus*, *Anaerobutyricum hallii*, and *Dorea longicatena*; each of these bacteria was elevated in the most obese participants (BMI ≥ 35 kg/m^2^). *Lachnospiraceae* are a diverse family of anaerobic and fermentative bacteria; they are particularly apt at utilizing varied long polysaccharides [54] as a source of energy. Their association with obesity has been observed in both case–control studies [55] and Mendelian randomization analysis [56], with the latter implying a causal effect. However, overall, it is difficult to denote this family as “harmful”, as they are major butyrate producers and display anti-inflammatory properties in multiple conditions (reviewed in [57]). Just as in diversity, associations might vary depending on the population. For instance, while a review by Chanda et al. [58] suggested mostly positive effects of *Blautia* in obese individuals, one of the studies with a negative effect came from a Polish population [59].

Differences in the abundance of bacterial groups result in changes in gut microbiota metabolic functions [60]. Of the metabolites generated by the intestinal microbiota, SCFAs are the most abundant [61]. Acetate, which is mostly produced by *Bifidobacteria* spp., maintains gut–epithelial barrier function and regulates intestinal inflammation, whereas butyrate acts as an important energy source for colonocytes and maintains gut barrier homeostasis [62,63]. Perhaps surprisingly, only minor alterations in SCFA profiles were found in this study, specifically for formic acid, and only in terms of its relative abundance. Increased abundance of formic acid might simply be a by-product of an unhealthy diet, as it was also higher in the overweight group than the control group. The overweight group consisted mainly of individuals from the esports group with a sedentary lifestyle [64]; soft and caffeinated drinks are a major source of formate [65]. Functionally, formic acid is an important substrate in methane production and may promote the growth of *Methanobrevibacter smithii*, which is associated with high body mass and obesity [66].

Other SCFAs were only weakly correlated with BMI; for butanoic and isobutyric acid this was a negative correlation. Butyrate plays a protective role against obesity (reviewed in [67]): experiments in mice suggested it influences multiple pathways and processes, including the enhancement of mitochondrial oxidative phosphorylation and the up-regulation of fatty-acid oxidation enzyme expression [68]. In addition, butyrate might help mitigate systemic inflammation, a characteristic of obesity, by inducing the differentiation of anti-inflammatory T-Reg cells [69,70]. By contrast, acetate was positively correlated with BMI in our study. Acetate influences the gut–brain–beta cell axis, which results in the development of metabolic syndrome and obesity in rodents [71]. However, other studies have found that acetate might decrease appetite levels [72]. Furthermore, unlike in our previous work [15], we did not observe a clear correlation pattern between metabolites and bacteria. Bacteria that correlated with the main SCFAs (propanoic, butanoic, and acetic acid) were both negatively and positively associated with BMI. This may point to an ambiguous role of SCFAs in obesity development, where they exert a protective effect as well as providing the host with additional energy and promoting fat tissue accumulation [8].

The functional changes related to obesity mirrored taxonomic shifts in our study, with more than 100 differential pathways, depending on the level of obesity. Most of the differential pathways concerned amino acid biosynthesis and energy generation (TCA cycle) and thus might simply be a reflection of large differences at the taxonomic level. Such changes were also observed previously in all classes of obesity [42]. Glucose, sucrose, and fatty acid degradation pathways were also overrepresented in the most obese patients (BMI > 35 kg/m^2^), which suggests a greater capability of obesity-related gut microbiota to metabolize simple dietary carbohydrates and fats. Again, this is far from surprising, as the first functional analysis of obesity-related human gut microbiota revealed differences in lipid and carbohydrate-related microbial metabolism [73]. Interestingly, the hexitol fermentation pathway was also overabundant in participants with a BMI > 35 kg/m^2^; this process results in the production of formate and might explain its increased abundance in the obese gut metabolome.

The human gut microbiome comprises at least 1800 genera and 15,000–36,000 bacterial species in low or high abundance that co-evolve with the host [74,75]; however, only a fraction of these bacteria are present in a single individual. Therefore, although defining healthy microbiome characteristics is crucial for health monitoring and devising personalized therapies that target the microbiota [21], creating a clear and generally accepted definition of a healthy gut microbiome has not been possible to date. Most of the previously proposed methods of defining a healthy microbiome (such as [19] or [21]) have been based on taxonomic composition. However, this strategy might prove less effective in obesity, as the microbiome related to this disease shows a large variance based on geographic location [76]. As mentioned above, in this study, we observed changes (higher diversity, for example) in obese people that might be a unique characteristic of the Polish population. By contrast, functional diversity is less pronounced in healthy microbiota [77], as the same biological processes can relate to different bacterial groups.

This suggests that more attention should be paid to the shift in microbial functional signatures, a good example of which is the cancer index we recently created, which is based on functional, rather than taxonomic, changes [78]. In a similar vein, Zielińska et al. [23] proposed a function-based measure to distinguish between healthy and dysbiotic microbiomes (the PDI, Predict Dysbiosis Index). In this study, we tested both taxonomic and functional indices, calculated in the same manner as in our previous work [15]. Both the PDI and the taxonomic index distinguished between people with extreme obesity and healthy controls. Our functional index also differentiated between people with lower grades of obesity and healthy controls. This suggests that subtle functional differences might be visible even at lower grades of obesity before they become more pronounced as BMI increases. Moreover, PDI lost its distinguishing power when taxonomic annotations were included. This might indicate that function-based indices are more robust in distinguishing between healthy and dysbiotic states of microbiota, especially if they are not caused by factors such as infection or associated with gastrointestinal symptoms (i.e., diarrhea). By contrast, no differences were observed between overweight participants and healthy controls, which potentially indicates that no permanent changes occur in the microbiome with weight gain.

## 5. Conclusions

Our study had several limitations, including gender imbalance within the esports cohort. Research indicates that the gut microbiome differs between males and females, likely due to hormonal differences and other factors influenced by sex. Therefore, lack of female representation could introduce bias in the study, potentially overlooking unique aspects of the gut microbiome related to female esports players. To address this, future studies should strive for more balanced gender representation within the esports cohort or account for sex as a confounding variable in the analysis. However, it is worth noting that our indices are based on associations adjusted for gender and age. A next significant limitation relates to the small size of the obesity I group (BMI 30–35 kg/m^2^), which may be not sufficient to draw strong conclusions about the relationship between BMI and other factors. Since underpowered studies have a higher risk of producing false negative results, failing to detect a real effect, further studies should increase the statistical power of the study by increasing the size of the milder obesity group, which refers to the BMI range of 30–35 kg/m^2^, making it more likely to detect real effects and draw more reliable conclusions.

We had limited access to patients’ health data, such as comorbidities. This was especially true for overweight and obesity class I individuals, who were recruited from healthy cohorts outside of the healthcare system. A lack of comorbidity data prevented further stratification of participants, which may have resulted in an underestimation of the differences in gut microbiota and its function between groups. We also targeted only a small number of metabolites, which may have resulted in missing significant changes in other metabolites. Nevertheless, we observed large obesity-related shifts in the microbiome on both taxonomic and functional levels, although taxonomic shifts might be geographic region-specific. Moreover, we demonstrated that changes in the microbiome related to obesity mostly concern individuals with a BMI of 35 kg/m^2^ or higher. This level of BMI is usually grounds for bariatric surgery in the presence of other comorbidities. Our results suggest that any treatment strategies targeting the microbiome in obesity, such as probiotics [79] or fecal microbiome transfer [80], might be effective only at higher degrees of obesity. Finally, we proved that a single microbial index can be used to distinguish between healthy and obese microbiota and that function-based indices are more useful for this task than taxonomy-based indices. Given the evidence we have presented on the potential utility of the “gut microbiome cancer index” [15] and the “gut microbiome obesity index” (this study), we believe that these innovative microbiome indices might be useful as part of routine metagenomics evaluations. However, studies have shown [81,82,83,84] that gut microbiome composition can vary based on geographic location, potentially due to environmental factors, local foods, and lifestyle. In addition, cultural practices, such as hygiene, food preparation methods, and social behaviors, as well as genetic background, can influence the gut microbiome. Therefore, an index developed in one geographical area might not be applicable to individuals in a different region. To improve the generalizability of the “gut microbiome obesity index”, it might be necessary to develop separate indices for different populations or geographical regions rather than relying on a single index. Furthermore, when developing or applying the index, it is crucial to consider the specific dietary patterns and cultural factors of the population being studied. Thus, future large-scale, multi-ethnic studies should involve diverse populations with comprehensive dietary and lifestyle data to identify universal and population-specific gut microbiome indexes.

## Data Availability

The dataset(s) supporting the conclusions of this article is/are available in the NCBI SRA at PRJNA1125836 and PRJNA885289.

## Supplementary Materials

The following supporting information can be downloaded at https://www.mdpi.com/article/10.3390/nu17142320/s1: Supplementary Table S1. Differential abundance of bacterial species in comparison between controls and esport and obesity groups. Supplementary Table S2. Differential abundance of bacterial species in comparison between controls and stratified obesity groups. Supplementary Table S3. Differential relative abundance of SCFAs and amino acids—comparison between control and esport and obesity groups. Supplementary Table S4. Differential relative abundance of SCFAs and amino acids—comparison between control and esport and two obesity groups. Supplementary Table S5. Differential relative abundance of SCFAs and amino acids—comparison between control and stratified obesity groups. Supplementary Table S6. Species associated with BMI. Supplementary Table S7. Species associated with BMI after correcting for age and sex. Supplementary Table S8. Functions associated with BMI after correcting for age and sex. Supplementary Figure S1. External functional and taxonomic health index (pHD) results, including correlation with BMI (A) according to Spearman’s rank correlation coefficient and differences between the control group and esports, overweight, and obesity groups (B–D).
